# How Does Embodying a Transgender Narrative Influence Social Bias? An Explorative Study in an Artistic Context

**DOI:** 10.3389/fpsyg.2020.01861

**Published:** 2020-08-04

**Authors:** Marte Roel Lesur, Sonia Lyn, Bigna Lenggenhager

**Affiliations:** ^1^Department of Psychology, Cognitive Neuropsychology With Focus on Body, Self, and Plasticity, University of Zurich, Zurich, Switzerland; ^2^BeAnotherLab, Barcelona, Spain

**Keywords:** embodiment, social cognition, naturalistic settings, art–science dialogue, sensorimotor sharing

## Abstract

Virtual reality (VR) protocols inducing illusory embodiment of avatars have shown a positive impact on participants’ perception of outgroup members, in line with the idea that the simulation of another’s sensorimotor states might underlie prosocial behavior. These studies, however, have been mostly confined to laboratory settings with student populations and the use of artificial avatars. In an interdisciplinary effort benefiting from the heterogeneous sample within a museum, we aimed at quantifying changes in interpersonal perception induced by embodying a transgender man narrating his life. We compared an artistic methodology mixing VR and elaborate sensorimotor stimulation to a more conventional primarily audiovisual VR experience. We tested how these affect embodiment and the perception of transgender men as measured by a brief implicit association test and a questionnaire. Neither significant difference in embodiment nor changes in implicit or explicit bias was found, the latter potentially due to the initially low bias in the group. We further assessed participants’ illusory embodiment as a function of age, finding a negative correlation between these. The results are discussed with respect to current theories of embodiment, differences between laboratory and real-life settings, and the intersection of art and science.

## Introduction

Human beings are individuals who are consciously aware of their selves and whose perception, cognition, and behavior are grounded in bodily processes (e.g., Dijkerman and Lenggenhager, 2018). Humans perceive themselves as a distinct physical entity, and experience the world from an embodied first-person perspective (Damasio, 1994). Yet, as a highly social species, humans can simulate and mimic other persons’ bodily states (Bernhardt and Singer, 2012); a process that arguably underlies social cognition, empathy, and prosocial behavior and thus might be core to society (Decety, 2010). Research has demonstrated that sensorimotor sharing [i.e., the interpersonal synchrony of the sensorimotor system at the phenomenal, functional, or neural level (Gallese and Sinigaglia, 2011; de Waal and Preston, 2017; Lamm et al., 2019)] and interpersonal perception are bidirectionally linked, such that humans tend to share more sensorimotor states with people they like and like people more with whom they share such states (van Baaren et al., 2009). Correspondingly, sensorimotor sharing is generally reduced for outgroup as compared to ingroup members (e.g., Avenanti et al., 2010). Recent work has argued that boosting sensorimotor sharing by exposing participants to a multisensory first-person perspective narrative/experience of an outgroup member might enhance empathy toward them (e.g., Galli et al., 2015; Maister et al., 2015). This process seems to rely on altering embodiment, the somatic awareness of the body, which is claimed to be grounded in the senses of body ownership and agency (Gallagher, 2000; Longo et al., 2008). Virtual reality (VR) techniques facilitate illusory embodiment through another’s first-person perspective together with the feeling of agency for and ownership of that person’s body (Petkova and Ehrsson, 2008; Roel Lesur et al., 2018), presumably maximizing sensorimotor sharing. Indeed, embodiment of a virtual outgroup member has shown to decrease implicit bias toward them (Peck et al., 2013; Maister et al., 2015; Banakou et al., 2016). Real-life scenarios in diverse samples would seem particularly relevant in social cognition investigations given the study matter. However, so far in behavioral research in general (Shamay-Tsoory and Mendelsohn, 2019), as well as in studies linking embodiment and social behavior (e.g., Peck et al., 2013; Maister et al., 2015; Banakou et al., 2016), these experiments have been mostly conducted in the laboratory, with a limited population of certain age range and socioeconomic background, which might bias the results (Henrich et al., 2010). In the current study, VR techniques were used, which have been argued to be beneficial to psychological research, because of their capacity in reproducing controlled stimulation settings of high ecological validity (Pan and Hamilton, 2018) outside the confined limits of a laboratory (Mottelson and Hornbæk, 2017). While artistic groups are applying VR methods derived from psychological research on the relation of embodiment and interpersonal perception in diverse settings across cultural boundaries (e.g., Bertrand et al., 2014, 2018; Sutherland, 2015), this study aimed at using these artistic applications to feedback to science. An artistic project using video-based VR with scientific measures from laboratory settings were combined to investigate in the context of a museum exhibition how social bias toward the transgender community might change after embodying a transgender man while listening to his personal story. While arguably a higher degree of ecological sensorimotor interactions from another’s perspective would maximize sensorimotor sharing, there seems to be no confirmatory evidence that more sensorimotor contingencies would result in a stronger reduction of bias compared to conventional (primarily audiovisual) VR. This comparison, however, could be important for potential applications of VR. The participants’ explicit and implicit bias toward transgender men was assessed before and after the exposure to an embodied, first-person perspective immersive video narrative (part of the Library of Ourselves; BeAnotherLab, 2020) shown on a head-mounted display (HMD). This experience was created by a group professionally working on enhancing empathy and prosocial behavior with focus on artistic methods (Sutherland, 2015; Bertrand et al., 2018). In two different groups, the influence of distinct degrees of multimodal synchrony of this experience on interpersonal perception was assessed. The audiovisual content was the same in both, but the presentation was either *conventional*, involving only vision, sound, and free head movements; or *sensorimotor*, that is, additionally involving enhanced somatosensory–motor interactions (i.e., synchronous tactile, motor, and proprioceptive signals). In the narrative, a transgender man talks about his life and journey of transformation. Building on the broad literature on multisensory stimulation and embodiment (e.g., Ehrsson, 2012) showing an important effect of multisensory coherences for illusory embodiment (Botvinick and Cohen, 1998; Kalckert and Ehrsson, 2012; Roel Lesur et al., 2018), a stronger embodiment illusion in the *sensorimotor* compared to the *conventional* group was hypothesized (hypothesis 1). Because experiencing an outgroup member’s story from a first-person perspective has shown to reduce bias toward the outgroup (Galli et al., 2015), and the content of the video-based experience has been developed to enhance empathy, reduced postexperience implicit and explicit biases in both the *conventional* and the *sensorimotor* group were expected, as compared to a *control* group, which had been subjected to an unrelated VR experience (hypothesis 2). Given the link between sensorimotor sharing with outgroup members and prosocial conducts toward them (e.g., Maister et al., 2015), a stronger reduction of bias toward the *sensorimotor* as compared to the *conventional* group was expected (hypothesis 3). Lastly, following evidence pointing at bodily self-plasticity in adults decreasing with age (Tajadura-Jiménez et al., 2012; Graham et al., 2015) and given the anticipated broad age range in the museum sample, a negative correlation between illusory embodiment with age was hypothesized (hypothesis 4).

## Materials and Methods

### Participants

A total of 71 individuals [age ranging from 20 to 72 years (mean, 34.1; SD = 13.3); 41 females] were recruited at a museum within the exhibition “100 Ways of Thinking” at the Kunsthalle Zurich^1^. They were assigned to a group with enhanced sensorimotor (i.e., involving touch, movement, and interaction with objects) stimulation [*sensorimotor* group, *n* = 49; mean age = 33.5 (SD = 12.0); 29 women] or to a group with conventional VR relying on audiovisual stimulation only [*conventional* group, *n* = 22; mean age = 35.3 (SD = 16.0); 12 women]. Both groups had free and visually contingent head movements. With the intention to record the maximum number of participants, more people were assigned to the *sensorimotor* than the *conventional* group because the shown art project was initially designed for such setting, and attendees showed more interest in it. All protocols were approved by the ethics committee of the Faculty of Arts and Social Sciences at the University of Zurich (approval no. 17.12.10). A total of 43 additional individuals (*control* group) were recruited at the University of Zurich in the framework of another study using a similar body illusion, which was unrelated to the theme of gender identity (unpublished work; the setup was comparable to Roel Lesur et al., 2020) as a control group [mean age = 22.40 (SD = 3.1); 32 women]. A Mann–Whitney comparison showed a significant difference of age between participants recorded at the museum and the laboratory (*U* = 2654, *p <* 0.001). Participants gave written informed consent to participate in the study, which was approved by the ethics committee of the Faculty of Arts and Social Sciences at the University of Zurich (approval no. 17.12.15).

### Materials

An Oculus CV1 HMD was used for stimulation. The software was designed using Unity 2017 for displaying a 235-degree prerecorded video portraying the first-person perspective as real person. It ran on a PC (Nvidia Geforce GTX 1080 8 GB; 16 GB RAM; Intel Core i7, 3.2 GHz). The video (see Supplementary Material) was filmed from the perspective of the transgender man using a Kodak SP360 4K camera at a resolution of 2,160 × 2,160 pixels at 30 fps; synchronous binaural audio recording of the environment was recorded from his ears using a Zoom H4 recorder and Roland CS-10EM microphones. Additional audio of his voice was recorded separately and superimposed to the environmental sounds. The questionnaires were displayed and answered using a computer monitor and a mouse; the tasks were programmed with JavaScript. Audio cues for the researchers appeared on headphones and were synchronized with the video.

### General Procedure

**FIGURE 1:**
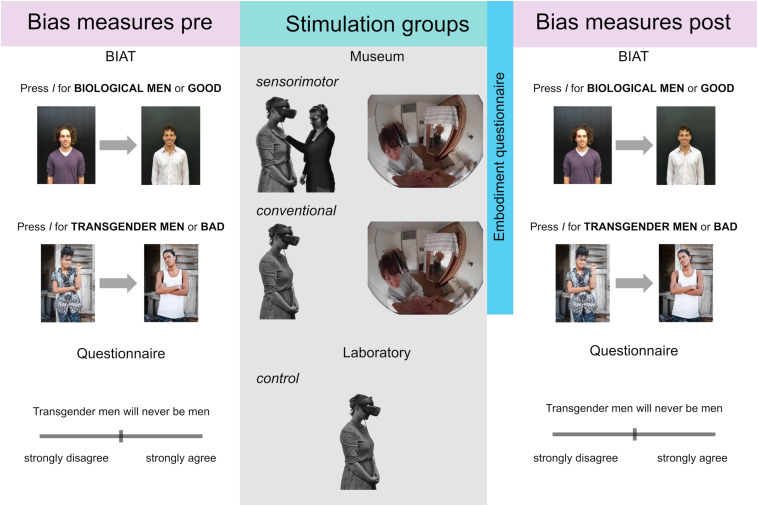
Experimental procedure showing the implicit and explicit bias tasks and groups differing by intervention. The *control* group was subject to an irrelevant body illusion. The images for biological men were comparable, but for the figure replaced due to image rights. The images for transgender men were taken from Gonzalez (2015) with permission from the author.

See Figure 1 for the general procedure in the three different groups.

### VR Experiences

#### Gender Identity Narrative Used at the Museum

The stimulation procedure at the museum consisted of displaying on an HMD an audiovisual narrative about Jonah, a transgender man talking about his life, friends, identity, relationships, and body transformation. Jonah’s story is told by himself and accompanied by different audiovisual environments of his hometown and close people shown from his perspective. The content duration is 7 min 47 s (see Supplementary Material for the content).

For the *sensorimotor* group, the feeling of the wind and interactions with objects and other people were reenacted by the experimenter; Jonah’s movements were reenacted by the participant. For example, when participants saw somebody touching their shoulder, they were touched by the experimenter to achieve multimodal synchrony (Figure 1), which has been described to induce a stronger sense of embodiment (Petkova and Ehrsson, 2008; Maselli and Slater, 2013; Roel Lesur et al., 2018). Stimulation was administered as follows: wind by manually fanning with cardboard, human touch with the experimenter’s hands, and self-touch by having participants touch themselves. Interactions with objects were facilitated as follows: using a chair to sit down, a smartphone to grab and touch, and a piece of cardboard (approximately corresponding to the size of a skateboard) to move with a foot. A reference to how these interactions were performed in another setting can be found in KCTS9 (2018). The experimenters wore headphones where instructions and timing for each action were presented to synchronize with the VR content. Additionally, they could visually monitor what participants saw in the HMD. Previous extensive rehearsal of the actions was performed.

The *conventional* group did not take part in the multimodal interaction, but sat down and experienced the audiovisual content presented in the HMD. They could still move their heads to explore the visual environment.

#### Irrelevant Body Illusion Used in the Laboratory Setting

For the *control* group, elements of this study were integrated into another embodiment-related experiment (similar to Roel Lesur et al., 2020) conducted at the University of Zurich in a laboratory setting. This group was included with the aim of disentangling the potential effect of retesting on both measures of bias. Participants in the *control* group were, similarly, subjected to a body illusion involving multimodal stimulation with the same HMD model; crucially, they were not exposed to any themes of gender identity or any narrative-based stimulation. The stimulation lasted approximately 30 min.

### Measures

Note that in order to prevent participant bias on the questionnaires due to the public setting in the museum, only they would look at the screen during the procedure and potential interruptions would be prevented by the experimenters.

#### Explicit Attitudes Toward Transgender

Before and after stimulation, participants were required to answer on a visual analog scale (VAS) presented on a PC an *Attitudes Toward Transgender Men and Women* questionnaire (Billard, 2018). Six of 12 items were selected that were answered on a scale ranging from strongly disagree (0) to strongly agree (100). The items were “transgender men… (1) will never really be men, (2) are only able to look like men but not be men, (3) are unable to accept who they really are, (4) are trying to be someone they are not, (5) are denying their DNA, (6) are unnatural.”

#### Brief Implicit Association Test

Participants’ implicit attitudes were assessed before and after exposure using a good-focal Brief Implicit Association Test (BIAT; Sriram and Greenwald, 2009) involving four categories of stimuli: a pair of contrasting attributes (positive vs. negative) and a pair of target images depicting a change in haircut of men (biological men) or a transition from a woman to man (transgender men; Figure 1). Note that while the pictures depict transgender women due to the availability of the images (Gonzalez, 2015), they included an arrow showing the transition in the other direction. The BIAT is a validated brief version of the well-known Implicit Association Test (Greenwald et al., 1998). Before the task, participants were given time to familiarize themselves with the images and their respective categories. The task was presented on a PC, and keystrokes were performed on a keyboard.

The BIAT consisted of six blocks in total, of which only two served as experimental blocks. In congruent blocks, participants were instructed to respond to images of biological men and positive attributes with the same keystroke (“I”). In incongruent blocks, transgender men were assigned the same key as positive attributes instead. Each block consisted of 16 trials.

D scores for each participant were calculated in line with Greenwald’s improved scoring algorithm (Greenwald et al., 2003), which incorporates data from the practice trials, uses a metric that is calibrated by each respondent’s latency variability, and includes a latency penalty for errors. Positive values represent a more positive association with biological men, and negative values with transgender.

#### Embodiment

For the two museum groups, directly after stimulation, participants were presented an embodiment questionnaire on a PC that was modified after previous studies assessing embodiment in similar experimental settings (e.g., Lenggenhager et al., 2007; Slater et al., 2010; Gonzalez-Franco and Peck, 2018). Dobricki and de la Rosa (2013) proposed question categories after a principal component analysis, and Gonzalez-Franco and Peck (2018) after a qualitative analysis; thus, we considered the inclusion of a question related to relevant categories to suffice for this study, given the limited time we could ask of participants attending the exhibition. The participants answered with a mouse on a VAS ranging from 0 (strongly disagree) to 100 (strongly agree) to six statements, of which three referred to illusory embodiment (“Sometimes it felt like the seen body is my own body,” “Sometimes it felt like I was in control of the virtual body,” “Sometimes I felt connected with the seen body,” respectively), one to the sense of presence (“Sometimes I felt like I was actually there in the presented environment”), one to enjoyment of the illusion (“I found the experience enjoyable”), and one to the perceived multisensory synchrony of the event (“The seen events were synchronous to the felt events”; low scores were expected in the *conventional* group as visual and somatosensory–motor stimulation would not correspond except for head movements).

The *control* group also completed an embodiment questionnaire within the unrelated experience; however, these data are not analyzed as they are not relevant to the research questions.

#### Data Treatment and Statistical Analysis

Statistical analyses were conducted using JASP (version 0.11.1); the data were tested for normality, and corresponding tests were performed. An aligned ranks transformation analysis of variance (ANOVA) for nonparametric factorial analyses (ART–ANOVA; Wobbrock et al., 2011) and the corresponding *post hoc* comparisons were performed using R version 3.5.1. Two-tailed comparisons are reported in all cases.

## Results

### Effect of Multisensory Stimulation on Embodiment in the Immersive Experience

**FIGURE 2:**
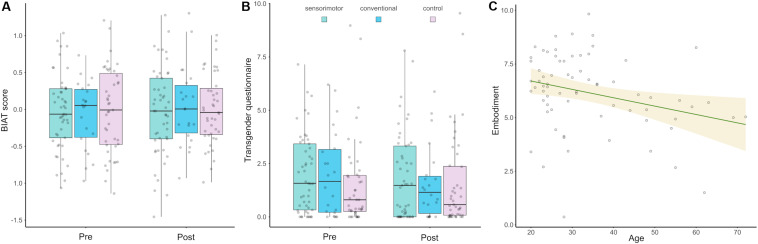
Medians, IQR, and participants’ mean scores for **(A)** the implicit measure and **(B)** the explicit measure, before and after the intervention for the three tested groups; and **(C)** negative correlation between participants’ age and embodiment score with linear regression and confidence intervals for the museum sample only (which includes both the *sensorimotor* and *conventional* groups).

In Table 1, the questionnaire scores and Mann–Whitney independent-samples *t* test statistics are depicted. Unlike predicted in hypothesis 1, no significant differences of embodiment or enjoyment between the museum groups were found; however, a trend was found for embodiment. A marginal difference of presence and a highly significant difference of perceived synchrony were found, with higher scores in the *sensorimotor* as compared to the *conventional* group. As embodiment and sensorimotor sharing have previously been shown to depend on age (Tajadura-Jiménez et al., 2012), and embodiment did not significantly differ between the two groups, a Spearman correlation was calculated for the whole museum sample. As predicted in hypothesis 4, age was negatively correlated with embodiment (ρ = −0.27, *p* = 0.023; Figure 2), suggesting that embodiment was stronger in younger participants.

**TABLE 1:** Median and IQR (given that data were not parametric) of the embodiment scores, Mann–Whitney comparisons between groups and rank biserial correlation (*rB)* as a measure of effect size are shown.

Variable	Sensorimotor group	Conventional group	*W*	*p*	*rB*
Embodiment	6.57 (2.07)	5.9 (2.54)	693	0.057	0.285
Enjoyment	6.8 (3.7)	5.75 (5.85)	673	0.097	0.249
Presence	6 (3.2)	5 (3.97)	698	0.049	0.294
Synchrony	7.3 (3.9)	3.6 (3)	828	<0.001	0.536

### Effects of Embodying Jonah on Bias Toward Transgender People

Further explorative analyses can be found in the Supplementary Material.

*Implicit bias*: An ANOVA with the within-factor group (*sensorimotor*, *conventional*, and *control*) and between-factor time (pre, post) was performed to assess the effect of the intervention on the BIAT scores (pre: mean = -0.034, SD = 0.52; post: mean = -0.004, SD = 0.54). No significant effects were found for time [*F*(1,111) = 0.47, *p* = 0.5, η = 0; hypothesis 2], group [*F*(2,111) = 0.04, *p* = 0.96, η = 0], or the interaction between these [*F*(2,111) = 0.18, *p* = 0.83, η = 0; hypothesis 3].

*Explicit bias*: For the questionnaire (pre: median = 1.25, IQR = 2.56; post: median = 0.9, IQR = 2.66; these scores are given to describe the nonparametric data), an ART-ANOVA with the within-factor group and between-factor time showed no main effect of time [*F*(1,111) = 3.5, *p* = 0.65, hypothesis 2], group [*F*(2,111) = 0.9, *p* = 0.39], or an interaction between these [*F*(2,111) = 1.2, *p* = 0.3, hypothesis 3].

## Discussion

### Multisensory Stimulation and Embodiment

In two groups pertaining to a sample taken during a museum exhibition, this study aimed to assess how embodying an outgroup member during either an enhanced *sensorimotor* VR experience involving movement and touch (*sensorimotor* group) or a more conventional audiovisual one (*conventional* group) would impact the perception of outgroup members. While a significantly higher embodiment score for the *sensorimotor* compared to the *conventional* group was expected (hypothesis 1), only a statistical trend in the predicted direction (together with a mild difference in presence) was found. These findings might be due to the reduced number of items in the questionnaire applied compared to other studies. However, while most studies suggest that congruent sensorimotor signals over fake bodies or limbs are fundamental to produce illusory embodiment (Botvinick and Cohen, 1998; Lenggenhager et al., 2007), it has been suggested that the induced substitution of optic flow related to head movements may be important for embodiment and even overwrite the effect of asynchronous signals in peripheral limbs (Roel Lesur et al., 2018). Here, both conditions had a congruent visual field, which might explain why only a trend was found in the data. Furthermore, while both the experimenter and participant attempted to synchronize their movements and the perceived synchrony scores were reasonably high, there might have been slight mismatches in the synchronization in the *sensorimotor* group, which may have affected the embodiment scores.

### Age and Embodiment

Interestingly, in line with hypothesis 4 and previous studies (Tajadura-Jiménez et al., 2012; Graham et al., 2015), across the full museum sample (including the *sensorimotor* and *conventional* groups), a negative correlation between age and embodiment was found, with illusory embodiment decreasing with age. Bodily self-plasticity is often measured as susceptibility to illusory embodiment; these results suggest that such plasticity decreases with age. However, other studies involving the rubber hand illusion suggest no differences between younger and older adults (Campos et al., 2018; Palomo et al., 2018). It could be argued that this decrease in embodiment with age is due to the different body appearance between elder participants and the young seen body; however, there is increasing evidence that it is possible to embody radically different bodies in terms of age (Tajadura-Jiménez et al., 2017) and other features (Hoort et al., 2011). It seems that investigations of bodily self-plasticity across different adult age groups have not generally used VR or full-body illusions. Given that the body goes through significant changes in late adulthood, bodily self-plasticity as an adaptive capacity is of relevance for fundamental and applied science. Thus, extending experimental settings to contexts other than university laboratories may be important, and VR might be an interesting tool to do so (Mottelson and Hornbæk, 2017).

### The Effect of Illusory Embodiment on Social Cognition

Unlike expected in hypotheses 2 and 3, the reduction of bias after as compared to before the transgender experience was not found in either the implicit or explicit measure for any of the experimental groups. This finding contrasts several studies showing differences in bias toward outgroup members after embodying an outgroup member (e.g., Peck et al., 2013; Maister et al., 2015; Banakou et al., 2016). Notably, in contrast to these, the current study administered a pre–post BIAT within the same session, and the results from an explorative analysis (see Supplementary Material) suggest that there may have been a practice effect. While similar designs have previously presented significant results (e.g., Galli et al., 2015), practice effects could have potentially masked the intervention effect in this study. Moreover, implicit association tests are not without pitfalls (Blair et al., 2001; Fiedler et al., 2006). An alternative potential explanation for the null findings could be that while previous studies based their stimulation on a visually salient skin color, here the identity of the embodied body was not revealed visually but through the story. Future studies should look at this important conceptual difference and how it affects social cognition. Importantly, other studies found no change of implicit bias after sensorimotor sharing with an outgroup member in a virtual setting (Hasler et al., 2014). The authors suggest that this may be related to the social setting presented in the virtual environment. Others found an increase of bias after embodying a dark-skinned avatar in a social setting (Groom et al., 2009), yet these results could also be related to a population bias regardless of the experimental manipulation. Future research linking narrative and social situations to explicit bodily conditions is encouraged to disentangle the potential mechanisms that may explain the contradicting results in the literature. In this study, a floor effect is plausible (see Supplementary Material) because of the generally low bias in the studied population previous to the intervention, which might have diminished the effects. Unsurprisingly, the population attending the exhibition and willing to take part in a study where they embody a transgender man had low explicit and implicit bias and might not be representative of the general population.

### Extending Science Through Alternative Spaces and Interdisciplinary Links

Following the above, performing this experiment in a museum setting does not overcome some of the problems intrinsic to doing research within universities (e.g., Henrich et al., 2010). Yet, the potential of VR to immerse participants in ecologically valid settings involving social situations may be still be important to further knowledge in several fields (Mottelson and Hornbæk, 2017). While methods that improve reproducibility are still needed, these types of collaborations between different disciplines and in diverse contexts may be important to move away from the artificial setting of laboratory studies. Although VR allows for a certain degree of reproducibility of the experience, the complexity of the social setting may always have an effect on participants’ expectations and behavior. A museum setting, as many other ecologically valid settings, is a socially active and complex environment with many parameters out of the researcher’s control. There is no clear escape from the paradox of maximally controlled settings versus ecological validity, yet VR may offer an important middle ground for future psychological research potentially benefiting from interdisciplinary collaborations. While future explorations in naturalistic settings are encouraged, they could benefit from finding public events attracting a more heterogeneous population, given that a museum might be similarly biased to a university setting.

Integrating artistic practices in scientific research might be beneficial to potentially expand scientific methods, as well as to validate certain aspects of the art methodologies (Borgdorff et al., 2020). However, as is often the case, in order to produce reproducible science, such methodologies might be subject to simplification and homogenization. Art, in contrast to quantitative science, tends to rely on qualitative approaches assuming the variability of participants’ interpretations and focus on the process rather than the outcome, potentially hindering scientific generalizations. Thus, in the transfer of artistic methods to scientific inquiry, important elements might be impaired without necessarily diminishing the legitimacy of artistic practice. For some artists, attempts aiming at the scientific legitimation of their work could imply an unwanted hierarchy between fields. Regardless of the focus of this work on experimental psychology, given its interdisciplinary nature, it seemed important to stress that potential benefits of art processes may be overlooked by the (however reproducible and robust) sometimes narrow lens of science.

## Data Availability Statement

The datasets generated for this study are available on request to the corresponding author.

## Ethics Statement

The studies involving human participants were reviewed and approved by Ethics Committee of the Faculty of Arts and Social Sciences at the University of Zurich (Approval Number: 17.12.10). The patients/participants provided their written informed consent to participate in this study. Written informed consent was obtained from the individual(s) for the publication of any potentially identifiable images or data included in this article.

## Author Contributions

BL and MR designed the experiment and wrote the overall content of the article. SL contributed with the data collection. BL, MR, and SL contributed to the statistical analysis and the results section. All authors contributed to the article and approved the submitted version.

## Conflict of Interest

The authors declare that the research was conducted in the absence of any commercial or financial relationships that could be construed as a potential conflict of interest.

## Footnotes

1 http://kunsthallezurich.ch/en/100-ways-thinking
