# Supplementary material for: How Does Embodying a Transgender Narrative Influence Social Bias? An Explorative Study in an Artistic Context
Source: Front Psychol. 2020 Aug 4;11:1861. doi: 10.3389/fpsyg.2020.01861 (PMC7417648; doi:10.3389/fpsyg.2020.01861)
Supplement: Supplementary file 1 [file Data_Sheet_1.docx]

**Supplementary explorative analyses**

**Implicit bias**

Given the initially low BIAT scores, a between-factor for low and high initial scores resulting from a median split was calculated. A Repeated Measures ANOVA with the within-factor group (*sensorimotor*, *conventional* and *control*) and between-factors time (pre, post) and initial bias (less preference for trans men, less preference for biological men) was performed. The initial bias factor showed a significant interaction with time (F(1, 108) = 34, *p < 0.001,* η = 0.08; see Supplementary Figure 1), but not with group (F(2, 108) = 0.18, *p* = 0.838, η = 0). Holm-corrected post hoc comparisons showed a significant difference between pre and post for low (*p* = 0.001) and high (*p* < 0.001) initial bias.

In order to assess the potential effect of age and embodiment in the BIAT scores, a repeated measures ANOVA for the museum sample (excluding the *control* group) with the within-factor time (pre, post) and between-factor group (*sensorimotor*, *conventional*) showed a main effect of time (F(1, 67) = 4.8, *p =* 0.03*,* η = 0.02)), but not of group (F(1, 67) = 0.02, *p =* 0.9*,* η = 0)), nor of the interaction of time and group (F(1, 67) = 0.88, *p* = 0.887*,* η = 0)). There was no effect of time when controlling for participant's age (F(1, 67) = 1.8, *p* = 0.18*,* η = 0.01)) nor embodiment scores (F(1, 67) = 3.7, *p =* 0.06*,* η = 0.08)) as covariates. Repeating the above procedure while excluding participants under the median of the initial BIAT score showed no main effect of time (F(1, 33) = 0.01, *p =* 0.9*,* η = 0)), group (F(1, 33) = 0.03, *p =* 0.86*,* η = 0)), nor of the interaction of time and group (F(1, 33) = 0.3, *p* = 0.59*,* η = 0)). There was no effect of time when controlling for age (F(1, 33) = 0.06, *p* = 0.81*,* η = 0)) nor embodiment (F(1, 33) = 0.27, *p =* 0.61*,* η = 0)) as covariates.

**Explicit bias**

Given the generally low initial questionnaire scores, an aligned ranks transformation ANOVA for non-parametric factorial analyses was performed comparing the difference between pre and post questionnaire scores for the between-factors group (*sensorimotor, conventional, control*) and initial bias (low and high, respectively below and above the median of the initial questionnaire scores). There was an interaction of group and initial bias (F(2, 108) = 6.38, *p* < 0.01; see Supplementary Figure 2). Holm-corrected Wilcoxon rank-sum comparisons showed a significant difference only for low initial bias between the conventional group and the control group (see Supplementary Table 1).

**Supplementary tables and figures**

Supplementary Table 1:

*Wilcoxon rank-sum comparisons between experimental groups of the questionnaire scores for participants with low and high initial bias respectively.*

| initial bias | comparison | *W* | *p* |
| --- | --- | --- | --- |
| low | sensorimotor - conventional | 200 | 0.71 |
| low | sensorimotor - control | 175.5 | 0.44 |
| low | conventional - control | 42.5 | 0.04 |
| high | sensorimotor - conventional | 70.5 | 0.44 |
| high | sensorimotor - control | 247.5 | 0.75 |
| high | conventional - control | 171 | 0.44 |


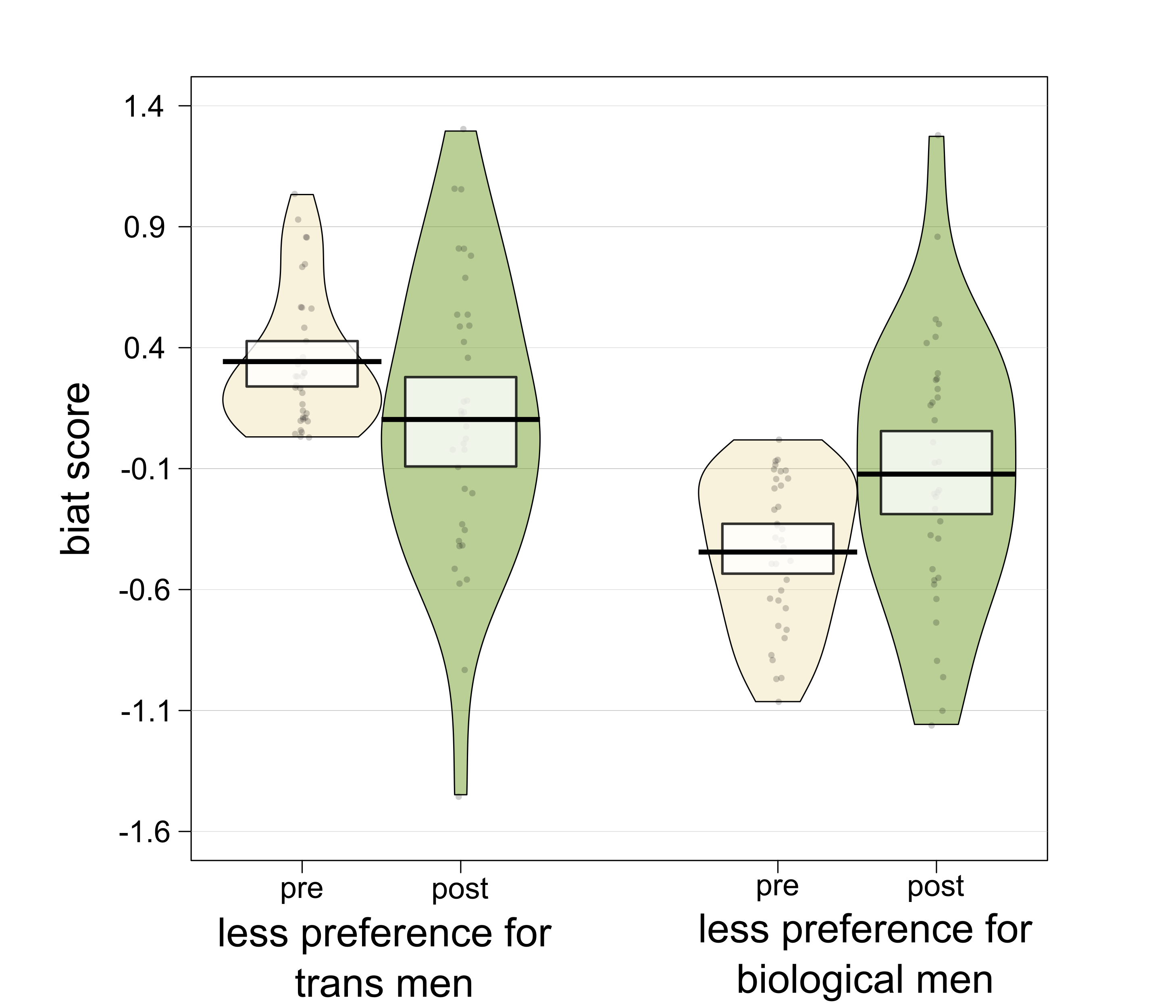


**Supplementary Figure 1:** Raw values, central tendencies and distribution of the BIAT score separated by initial bias (low, high) and the respective time (pre, post).


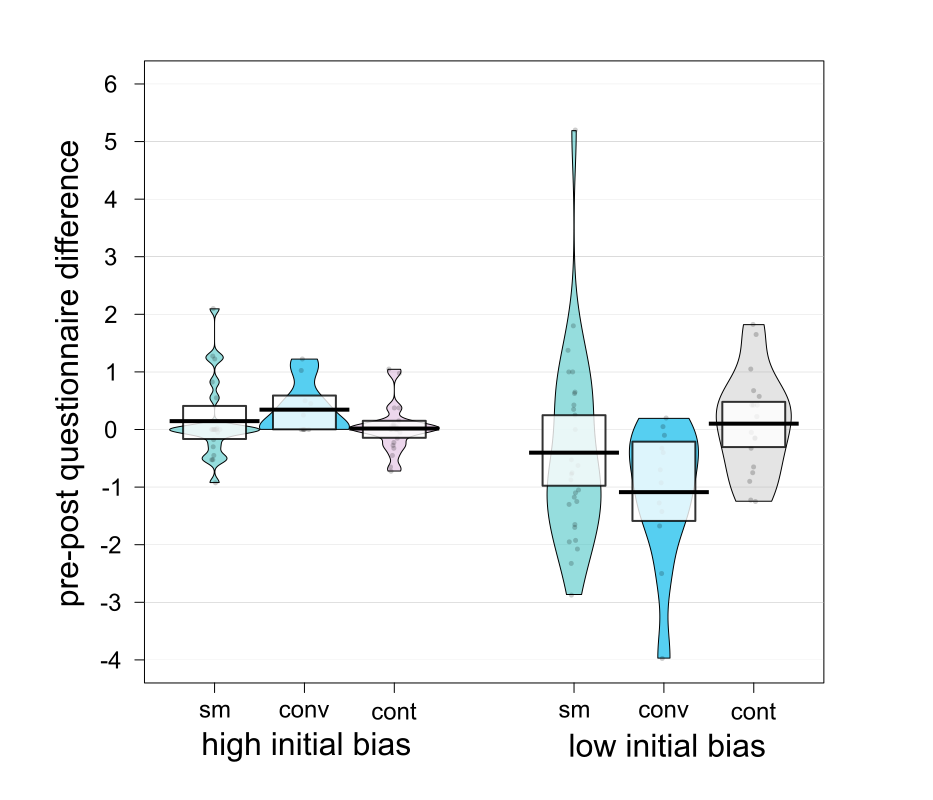


**Supplementary Figure 2:** Individual values, central tendencies and distribution of the pre-post difference of the questionnaire score separated by initial bias (low, high) and the respective group (sm = *sensorimotor*, conv = *conventional*, cont = *control*).
